# Radiographic Incidence of Lumbar Spinal Instability in Patients with Non-spondylolisthetic Low Backache

**DOI:** 10.7759/cureus.2420

**Published:** 2018-04-04

**Authors:** Ashok K Rathod, Rakesh P Dhake

**Affiliations:** 1 Department of Orthopaedics, LTMMC & LTMGH, Sion, Mumbai

**Keywords:** low backache, lumbar spinal instability

## Abstract

Introduction

Radiological lumbar spinal instability may exist without obvious spondylolisthesis. We aim to determine the incidence of this non-spondylolisthetic cause of instability in conservatively managed patients and operated groups of patients. We also attempted to study the relationship between instability and its occurrence with respect to age, sex, signs and symptoms.

Materials and methods

Twenty-three patients treated conservatively (group A) for non-spondylolisthetic backache were studied for radiological evidence of instability by evaluating angular rotation and sagittal translation at each lumbar motion segment. The influence of age, sex, signs and symptoms on the occurrence of instability was studied. A total of 18 patients treated surgically (group B) for non-spondylolisthetic backache in the form of discectomy/decompression were evaluated for occurrence of instability at three months, six months and nine months postoperatively.

Results

Four out of 23 patients (17.4%) in group A had radiological instability. Angular rotation was found to have negative correlation with age, while sagittal translation did not show any consistent correlation with age. Neither had any significant correlation with sex. The incidence of instability in patients treated with discectomy at three months and six months was 20% which reduced to 10% at nine months while that in patients treated with decompression was about 37.5% over three months, six months and nine months of follow-up.

Conclusion

If patients with spondylolisthesis were excluded from the study, instability could still result from the rotational component in sagittal plane. Secondary iatrogenic instabilities do result in patients undergoing extensive decompression for spinal stenosis and should always be thought of.

## Introduction

Low back pain is a prevalent medical problem with a significant proportion being of mechanical origin and is often referred to as instability. Estimates of the percentage of patients with low back pain arising because of spinal instability range from 13% to 30% of the total population of patients with mechanical low back pain [1].

The lumbar spine, although often described as a single functional unit, is composed of five vertebrae forming what are called "motion segments" connected in series [2]. Each motion segment consists of two adjacent vertebral bodies and the connecting ligament [3]. It is the smallest functional unit of spine exhibiting biomechanical characteristics similar to those of the entire spine [2]. During spinal movements, the adjacent vertebrae maintain their relationship with each other due to configuration, orientation and integrity of facet joint complexes, the integrity of various ligamentous structures and due to the highly specialized connective tissue structure, the intervertebral disc [4]. Translation and rotation can occur at each spinal motion segment during lumbar spine movements in any of the cardinal body planes [2]. The maintenance of stability of the lumbar spine during these movements requires the coordinated movements of multiple motion segments, and a lack of stability may potentially occur at any lumbar segment in either translational or rotational movements, or both [5].

Unfortunately, the traditional teaching about instability is generally restricted to spondylolysis and resultant listhesis to the extent that many of the surgeons even refuse to believe that anything else could be an instability [4]. In this study, we try to determine this little fraction of instability due to mechanical causes other than spondylolisthesis along with the incidence of secondary lumbar instability at the operated spinal segments following standard surgical procedures like discectomy and decompressive laminectomy in patients with no obvious spondylolisthesis or instability preoperatively.

## Materials and methods

A cross-sectional study with a prospective follow-up of 41 patients between 30 and 65 years of age visiting the outpatient clinic of our hospital due to low back pain with or without radiation into the lower extremities was conducted. They were subjected to a neutral anteroposterior (AP), lateral, flexion-extension and oblique X-rays of lumbo-sacral (LS) spine. Patients excluded were those who had a contraindication to radiographic assessment (e.g., pregnancy), spondylolisthesis, previous lumbar surgery, spinal trauma, spinal tumors including metastasis, osteoporosis with compression fractures, or unable to actively flex and extend the spine adequately due to pain or muscle spasm. All procedures performed were in accordance with the ethical standards of the institutional research committee. Informed consent was obtained from all individual participants included in the study. The patients were divided into two groups:

Group A (treated conservatively) included 23 patients (13 males and 10 females) with a mean age of 46.7 years. The mean duration of symptoms was 6.3 months. These patients were treated conservatively with analgesics, physiotherapy, back and abdomen strengthening exercises, lumbar traction, etc. at the time of evaluation and may or may not have required surgery in future. This group with low backache, not due to spondylolisthesis, was studied to determine the prevalence of instability.

Group B (operated) included 18 patients (11 males and seven females) with a mean age of 47.4 years who were operated for low backache with no radiographic evidence of preoperative spondylolisthesis or instability. Patients with revision surgery or other lumbar surgical procedures in past were excluded. Out of 18 patients, 10 patients had a prolapsed intervertebral disc and were treated with standard fenestration lumbar discectomy. The remaining eight patients had stenosis of lumbar canal which was treated with decompressive laminectomy. The indication for surgery of lumbar disc herniation at our clinic was persistent severe sciatica after at least six to eight weeks of conservative treatment and a magnetic resonance imaging (MRI)-verified disc herniation that correlated to the clinical picture.

Anteroposterior, lateral neutral and lateral flexion-extension X-rays were obtained in standing position. Considering the low back pain, patients were asked to move their trunk as much as they could. Radiographs judged to be unsuitable for measurement were those that implied a plane of radiographic projection not parallel to the lumbar segments, exhibiting a double vision of the vertebral edges. Radiographic instability at lumbar segments based on three lateral radiographs of neutral, flexion and extension was assessed by following three variables:

a) Anterior slip of superior vertebrae on inferior vertebrae (SN) on lateral X-ray in neutral or resting position

b) Segmental angular rotation (AR)

c) Sagittal translation (ST)

These three parameters have been used previously by many for radiological analysis of segmental instability in sagittal plane [6-8]. We used them to assess the radiological instability in Group A patients at the time of the first visit in the outpatient department. Group B patients were assessed at three months, six months and nine months after surgery.

**Figure 1:**
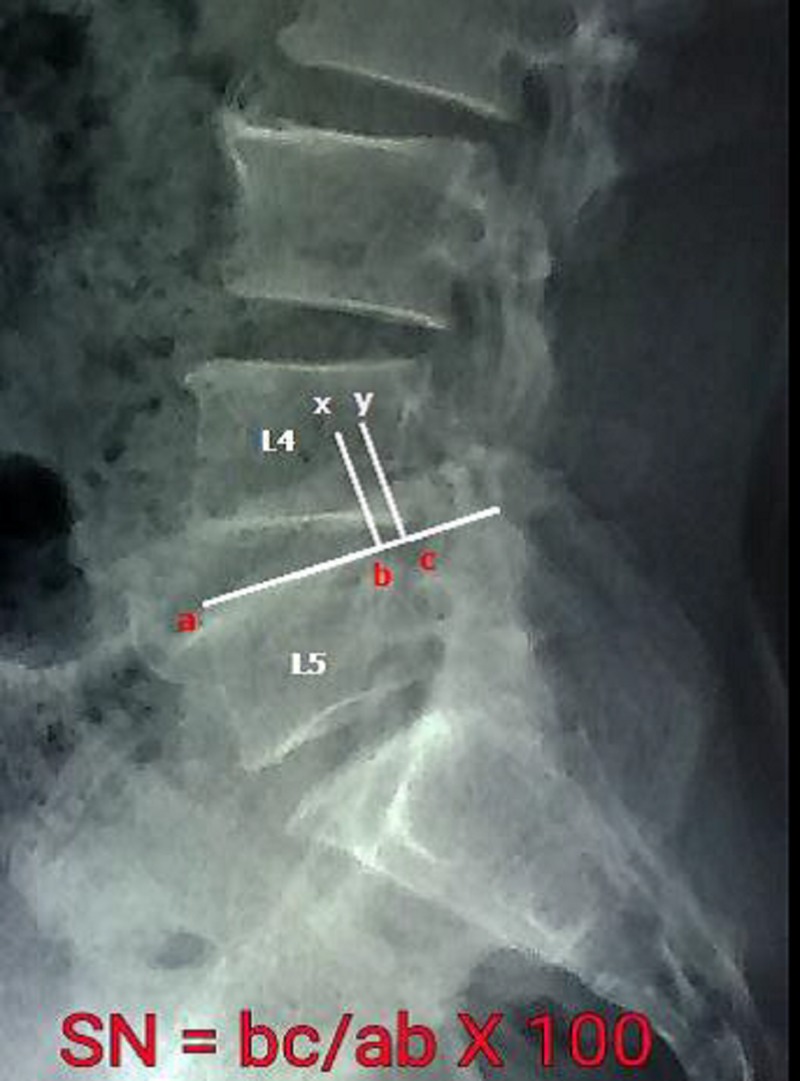
Method of measuring anterior slip in neutral or resting position (SN).

The measurement of each slip was performed using methods described by White and Panjabi [2], which include three landmarks for measurement: for example, at L4-L5 segment, it includes the anterior edge and the posterior edge of the upper endplate of L5 and the inferior posterior edge of L4. It was considered that a less variable result could be obtained by this system than with other methods using more than three landmarks [9]. A baseline is drawn passing the two landmarks at the anterior and posterior vertebral edges at the superior endplate of L5. Two lines are drawn perpendicular to the baseline passing the two landmarks at the posterior inferior edge of L4 and superior posterior edge of L5 vertebrae. The distance between the two lines is obtained on each, flexion and extension film. Slip in neutral position (SN) is calculated by the distance between these two lines on lateral X-ray in neutral position as shown in Figure 1.

**Figure 2:**
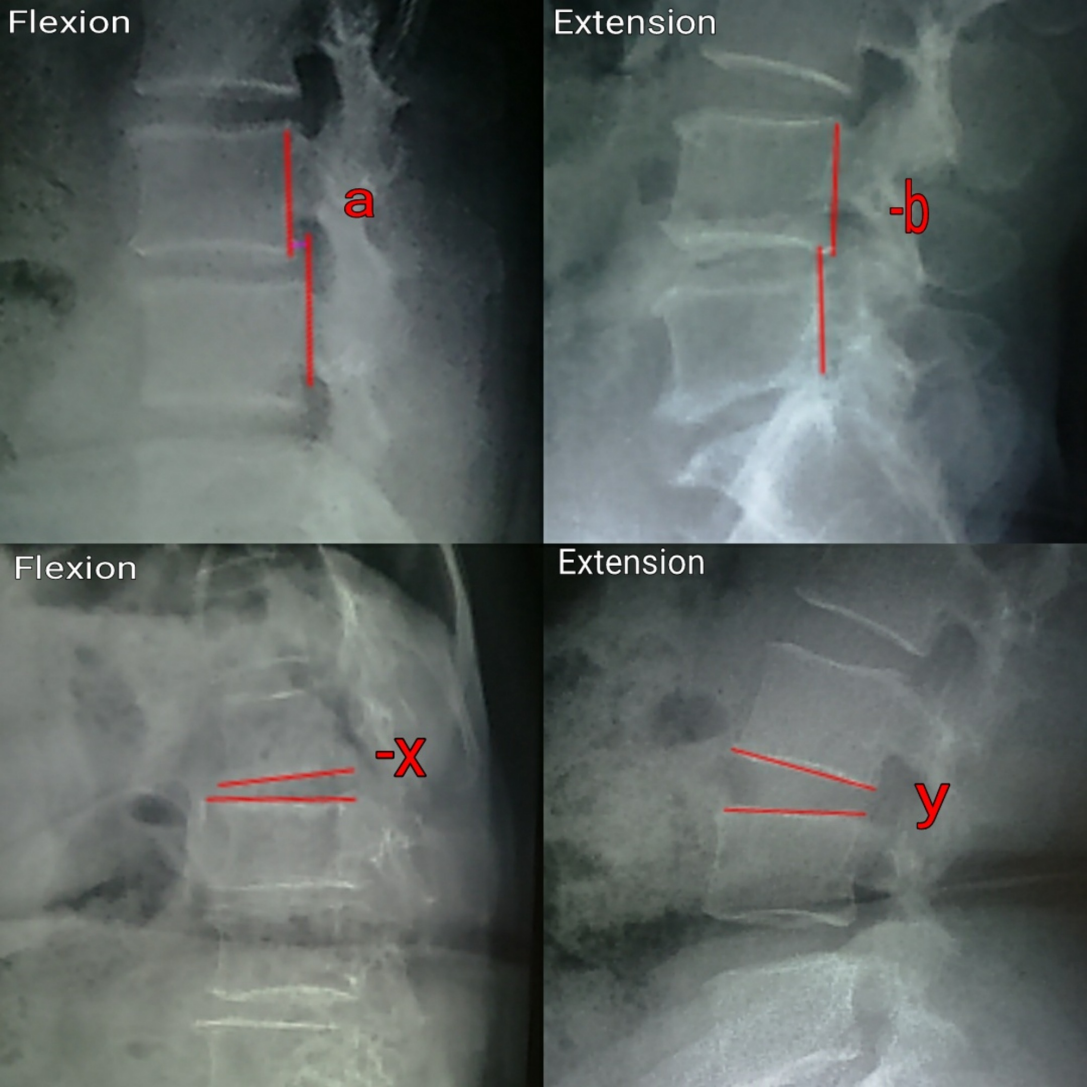
Method of measuring angular rotation (AR) and sagittal translation (ST).

The amount of sagittal translation (ST) was obtained as the difference between displacement on flexion and extension X-rays. Forward displacement of the superior vertebrae in flexion is assigned a positive sign and the backward displacement in extension is given a negative sign. Sagittal translation in Figure 2 below can thus be calculated as ST = a-(-b).

The magnitude of the translation at each segment was calculated by measuring the displacement of the superior vertebra as a percentage of the vertebral body width measured at superior endplate of the inferior vertebra. This method avoids inaccuracies related to magnification [10]. The slip and the translational measurements were calculated directly on the X-ray films.

End-plate angle was defined as the angle generated by a line drawn from the inferior margin of the superior vertebral body and another line drawn from the superior margin of the inferior vertebral body. Angular rotation (AR) was defined as the difference between the end-plate angle obtained from the extension film and that obtained from the flexion film. All the angular rotations were measured with the help of a computer-based software. The backward angulation on the extension was given a positive sign and forward angulation on the flexion was given a negative sign. The difference of the intervertebral angles between flexion and extension films was measured. Angular rotation in Figure 2 above can thus be calculated as AR = y-(-x).

We did not consider slip of vertebrae on lateral X-ray in neutral position (SN) in group A because we had already excluded patients with spondylolisthesis on resting lateral X-ray. Hence, the value of SN in this subjects of group A would be very minimal or zero. However, the parameter of slip in neutral position (SN) was considered in group B patients in order to look for any iatrogenic insult to the posterior structures that resulted in an anterior slip. Thus, group B patients were evaluated for instability on the basis of three parameters – slip in neutral position (SN) as on resting lateral X-ray, and angular rotation (AR) and sagittal translation (ST) as calculated on lateral flexion and extension X-rays.

The following criteria given by White and Panjabi [2] for determining rotational or translational instability were applied at each motion segment:

a) Angular rotation of >15 degrees at L1-L2 to L3-L4 segments, >20 degrees at L4-L5 segment and >25 degrees at L5-S1 segment, and/or

b) Sagittal translation of >4.5 mm or 15% of vertebral body width.

The patient was thought to have instability if any of the motion segment crossed the thresholds mentioned above. All computer-assisted statistical tests were calculated using SPSS software. p-value < 0.05 was considered as the level of significance.

## Results

Group A (Non-operated/pre-operative)

Out of 23 patients in the non-operated group A population, four patients (17.4%) had an evidence of radiological instability which was mainly due to the angular rotation and none had an instability caused by sagittal translation. The incidence of instability at each of the motion segments along with respective mean angular rotation and sagittal translation is summarized in Table 1. L4-L5 segment had the highest amount of mean angular rotation and sagittal translation.

**Table 1:** Table showing mean segmental angular rotation (AR) and sagittal translation (ST) along with incidence of angular instability and translational instability at each motion segment.

Motion segment	Mean AR (degrees)	Mean ST (% of vertebral body width)	Angular instability (% of Group A subjects)	Translational instability (% of Group A subjects)
L1-L2	8.74	0.78	4.4	0
L2-L3	7.87	0.81	4.4	0
L3-L4	7.96	2.47	8.7	0
L4-L5	9.17	2.87	8.7	0
L5-S1	8.4	0.8	0	0

On correlating the angular rotation and sagittal translation with age using the Pearson’s coefficient (r-value) we found the ‘r’ value to be negative for all the five lumbar segments which signifies that excessive angulation was prevalent in younger age group and it decreased as the age progressed. Thus the younger population had a higher angular rotation as compared to the elder. The mean age of population with excessive angular rotation was 41.75 years. However, sagittal translation did not show any consistent correlation with age (Table 2).

**Table 2:** Table showing correlation of age with angular rotation (AR) and sagittal translation (ST) at each of the motion segment.

Motion segments	L1-2		L2-L3		L3-L4		L4-L5		L5-S1	
	AR	ST	AR	ST	AR	ST	AR	ST	AR	ST
Pearson coefficient (r-value)	-0.34	-0.27	-0.57	-0.031	-0.36	0.48	-0.33	-0.08	-0.46	0.002
p-value	0.1	0.2	0.004	0.89	0.084	0.021	0.12	0.71	0.027	0.99

We also compared angular rotation and sagittal translation with sex using non-parametric method (Mann-Whitney Test). We did not find any significant correlation between sex and angular rotation or sagittal translation except at L1-L2 segment which showed angular rotation to be more in males ( p-value = 0.017) (Table 3).

**Table 3:** Table showing correlation of sex with angular rotation (AR) and sagittal translation (ST) at each of the motion segment.

	L1-L2		L2-L3		L3-L4		L4-L5		L5-S1	
	AR	ST	AR	ST	AR	ST	AR	ST	AR	ST
Man-Whitney U	26.5	62	47	63	45.5	43.5	64.5	62.5	51.5	56
p-value	0.017	0.77	0.25	0.86	0.22	0.13	0.97	0.86	0.4	0.34

As the sample size was small, Fisher’s exact test was performed to compare the occurrence of instability with respect to sex and various spinal symptoms and signs separately. We found that occurrence of instability had no significant correlation (p > 0.05) between sex and symptoms of the patient (Table 4).

**Table 4:** Table showing correlation between occurrence of instability with sex and various spinal signs and symptoms.

		Instability	No instability	p-value
		(n = 4)	(n = 19)	
Sex	M	2	11	0.396
	F	2	8	
Radiation	+	4	16	0.547
	-	0	3	
Claudication	+	4	13	0.268
	-	0	6	
Tenderness	+	2	12	0.369
	-	2	7	
Spasm	+	0	8	0.154
	-	4	11	
List	+	1	1	0.3
	-	3	18	
Ext catch	+	1	5	0.46
	-	3	14	
RTS	+	2	4	0.23
	-	2	15	

Group B (Post-operative)

Out of the 18 patients operated for non-listhetic backache, five had an instability at three months and six months (27.8%). The incidence got reduced to four patients having instability at nine months (22.2%).

We calculated the instability contributed by type of surgery separately at three months, six months and nine months. The incidence of instability in patients treated with discectomy at three months and six months was 20%. However, at nine months the incidence got reduced to 10%. Out of two patients who showed to have instability following discectomy at three and six months of follow-up after surgery, one patient operated at L5-S1 segment showed reduction in the angular rotation at the operated segment. The angular rotation was reduced to less than the cut off value for instability at nine months and thus only one patient operated with discectomy (out of 10) had instability at nine months following surgery. The incidence of instability in patients treated with decompression was about 37.5%. It did not show any change over three months, six months and nine months of follow-up.

On comparing the three parameters SN, AR, and ST over three months, six months and nine months for discectomy and decompression separately using Friedman test (non-parametric method), we did not find any significant difference (p-value > 0.05) except the SN at L4-L5 level in the discectomy group and SN at L1-L2 level in the decompression group. The following table demonstrates the value of significance at each level with respect to all the three parameters (Table 5).

**Table 5:** Comparison of the three parameters: angular rotation (AR), sagittal translation (ST) and anterior slip in neutral or resting position (SN) over three months, six months and nine months in patients treated with discectomy and decompression, separately, at each motion segment.

	L1-L2			L2-L3			L3-L4			L4-L5			L5-S1		
	SN	AR	ST	SN	AR	ST	SN	AR	ST	SN	AR	ST	SN	AR	ST
Discectomy	0.36	0.26	0.36	0.86	0.56	0.36	0.36	0.82	0.15	0.001	0.35	0.36	0.67	0.56	0.49
Decompression	0.001	0.13	0.36	0.36	0.66	0.6	0.36	0.43	0.17	0.66	0.96	0.66	0.36	0.66	0.6

## Discussion

Researchers have used various criteria for identifying abnormal kinematics in groups of patients with low back pain, with the most common criteria being radiographically measurable abnormalities in the magnitude of sagittal plane rotation and translation. To date, however, there has not been a consensus among authors regarding either the methodology for measuring motion, or the cut-off value beyond which the motion segment should be diagnosed as having a lumbar segmental motility disorder [11].

It has been proposed that aber­rant motion and dysfunction from struc­tural lumbar segmental instability exist not only at end range but during midrange spinal movements, which these tests might not identify. Flexion-extension radiographs simply assess vertebral displacement statically at end range [9,12] which, theoretically, would only detect the function of the pas­sive stabilizing subsystem [13]. This might have significant limitations in detecting dysfunction from structural lumbar segmental instability that oc­curs within the neutral zone (midrange spinal motion) [14].

Also, If a patient is unwilling to flex or extend fully from a standing position, perhaps because of pain, fear, or apprehension, both rotation and translation values will be low, even if the patient's spine was actually capable of moving normally [15]. This type of guarding behavior may mask lumbar segmental instability, leading to a false negative finding. Despite these limitations with functional radiographs, it has become the standard for evaluation of instability because of its simplicity, low expense, and wide availability.

Group A (non-operated)

Our findings support some previously hypothesized relationships and fail to support others. We found patients with radiographic instability (mainly due to angulation) were younger, supporting the theorized etiological sequence of stabilization following a period of instability in patients with low back pain. It is important to correlate clinically, since many asymptomatic patients may have spondylolisthesis or radiographic instability, with sagittal angulation as high as 25° being reported in healthy volunteers [16]. Even when present, abnormal movements are not necessarily the cause of pain. The abnormal and irregular transfer of loads and their concentration through spinal joints would in many cases be the real cause of mechanical pain, just as in other joints in the body [17].

The relationship between the three radiological parameters considered (SN, AR and ST) and symptoms have been reported recently, however expecting spondylolisthesis, little information has been obtained [7,18,19]. We calculated the mean angular rotation and mean sagittal translation at each segment separately. Both the parameters were found to be highest at the L4-L5 segment. These findings are in accordance with Golbakhsh et al. [20] who evaluated translations and angulations for each level separately in their study of correlation between pelvic incidence and lumbar instability. They observed most translations and angulations occur at L4-L5 level followed by L3-L4 level.

Before Iguchi et al., the relationship between age and the three radiological factor was not well established except in cases of spondylolisthesis [21]. They concluded that the presence of patients with excessive angulation and translation in younger age groups suggests they have a hypermobile segment with least degenerated discs. On correlating age with angular rotation (AR) using Pearson coefficient (r-value), we found the r-value to be negative which suggest that angular rotation was higher in younger subjects and lower in elders. This finding of angular rotation is well in concurrence with that of Iguchi et al. However, sagittal translation did not show consistent relationship with age at all segments in our study. These variations in the results of sagittal translation may be accounted to the small number of patients and exclusion of spondylolisthetic patients.

Despite the paucity of information about the relationship between segmental instability and disc degeneration and lumbar symptoms [19], increasing age is generally accepted to have a greater influence on disc degeneration [8], and degeneration has also been correlated with a lower disc height [8], and smaller sagittal angulation. Kaigle et al. [22] demonstrated that there was always less movement in the degenerated spine as compared to normal subjects. It may be argued that, unfortunately, this reduction of movement is associated with abnormal patterns of movement, and this is the meaning of “instability”. However, despite considerable efforts over many years, using flexion/extension films, no clear relationship has been established between pain and such abnormal movements. In other words, patients with degenerative disc disease may exhibit abnormal patterns of movement, yet have no pain [17].

We compared angular rotation and sagittal translation at each segment with sex. Men and women did not show any significant difference except that the angular rotation at L1-L2 segment was more in males. These findings, in concurrence with others [23], suggest that there is no significant difference in these kinematic variables between the sexes.

It is important to note that anteroposterior sliding of the fifth lumbar vertebra on the sacrum can hardly take place at any time, despite disc degeneration, because the articular processes between the fifth lumbar vertebra and the sacrum tend to look forwards and backwards (coronal plane), whereas in the other lumbar vertebrae the articular surfaces look outwards and inwards (parasagittal plane), a disposition that allows anteroposterior displacement if there is instability from disc degeneration [24]. Even when gross disc degeneration is demonstrated radiologically at the lumbosacral level, instability seldom exists, unless there is a subluxation at the apophysial joints from osteoarthritic degeneration or increased obliquity of the facets, or unless the facets tend to lie in the parasagittal plane instead of in the coronal plane. Such instability, if it exists, is never so great as at the other lumbar intervertebral joints. In our study, we did not find any angular or translatory instability at L5-S1 segment.

The mean angular rotation and mean sagittal translation was found to be highest at the L4-L5 segment as mentioned in the results. Tamrakar et al., in their study on radiological evaluation of lumbar instability, found a more sagittal orientation of facet joints at L4-L5 level in the degenerative spondylolisthesis group, when compared with the normal group. This sagittal orientation facilitates vertebral slippage when the other predisposing factors are present. They stated that because of these abnormalities and the preponderance of coronal orientation of the L5-S1 facet joints, the majority of degenerative spondylolisthesis occurs at the L4-5 level.

The overall incidence of instability in four patients out of 23 (17.4%) in group A is well in accordance with those mentioned in the literature. However, the comparatively less percentage of instability in our study could be due to the fact that we had excluded the patients who had spondylolisthesis. Thus, instability attributed to spondylolisthesis was not considered.

Group B (Operated patients)

Iatrogenic instability following lumbar surgery is more often seen than is commonly believed [4]. Different instability patterns, singly or in combination, may be seen even in younger patients following extensive decompression for spinal stenosis. In post-operative patients, the scar tissue may get stretched, the muscles may lose their endurance, strength, agility and the patient may be forced to give up the cautious care of his back. Either as a matter of single acute stressing during an inopportune moment or as a matter of chronic local stressing, this state of occult instability would manifest itself clinically [4]. In a study conducted by Iida et al. [25], they concluded that in patients under 60 years of age, instability at the operated level tended to appear in cases of wide laminectomy more often than in cases of partial laminectomy. Occurrence or progress of instability seems to be promoted by resection of the posterior spinal elements rather than the disc. Hakelius also stated that wide laminectomy tends to result in lumbar spinal instability [26].

Lumbar disc herniation is a manifestation of degenerative disc disease, which we believe should mainly be linked to stage one or two of the Kirkaldy-Willis concept of three stages of the degenerative process stated previously [27]. Mobility, which in our study was evaluated as inducible motion on flexion and extension, can be anticipated to decrease should the process progress to a fixed deformity. According to these theories, patients with lumbar disc herniation will develop decreasing mobility of the affected disc segment over time, regardless of whether there has been any surgical treatment. The influence of the surgical procedure is unclear [27].

The incidence of instability in 10 patients treated with discectomy at three and six months in our study was 20% which got reduced to 10% at nine months as one patient showed a gradual reduction in the angular rotation at the operated segment over the period of follow-up. No patient treated with discectomy had listhesis on neutral standing X-ray and none had an instability due to translational component. However, in a study conducted by A. A. Mascarenhas the incidence of radiological instability in 83 patients treated with discectomy was 34.9% out of which 19.2% had angular rotational instability and 15.7% showed sagittal displacement. This significant amount of sagittal displacement in their study might be due to an iatrogenic damage to the par interarticularis.

In our patients treated with decompressive surgeries, the decompression was achieved with minimal resection of the central lamina, removal of the thickened ligamentum flavum with or without removal of the disc depending on whether it was herniated or not. In all these cases there was good margin left between the resected lamina and the pars interarticularis. Facetectomy was not done in any of the cases.

The presence of large osteophytes on the X-rays around the narrow degenerated disc shows an attempt on the part of the body to stabilize the unstable segment. Contrary to the common belief, these osteophytes do not in any way indicate that the segment is successfully stabilized or even sufficiently stiffened. Patients of segmental instability with sufficient indication of pre-operative stabilizing attempt often have further instability after a posterior decompressive procedure. In our study, we attempted to diagnose such occult instability and newly created instability due to an iatrogenic insult to the posterior structures. Out of the eight patients who were treated with decompression, three patients (37.5%) demonstrated evidence of radiological instability at the operated segment. Out of the three patients, one patient had an instability due to slip in neutral position (SN > 15% of vertebral body width) along with rotational instability in sagittal plane. The other two patients had pure angular rotational instability. No patient had an instability due to translational component on flexion-extension X-rays.

A cadaver specimen study showed that total discectomy resulted in significantly more angular motion in all directions when compared to subtotal discectomy [28]. In a clinical study with biplanar radiography before and three months after discectomy, the authors concluded that discectomy with minimal resection of the laminae did not produce instability but follow-up was restricted to three months [29]. Thus, the long-term effects of a lumbar discectomy on segmental stability are not known [27].

We followed up the patients for nine months postoperatively to observe the effect of surgery on motility of the operated segment. Angular rotation and sagittal translation at three, six and nine months did not show any significant difference in both decompression and discectomy patients. However, one patient treated for discectomy at L5-S1 segment showed a gradual reduction in AR at the operated segment, namely L5-S1, reaching below the cut off value for instability. Although this observation is similar to that of Halldin et al., who found changing patterns of inducible displacement at L5-S1 level, it needs to be studied further on a larger population operated at L5-S1 segment and further research is required to conclude how the pattern of mobility at L5-S1 segment differed from other lumbar segments post-operatively.

Due to this one patient who showed decreasing trend in the angular rotation on follow-up, the incidence of instability got reduced from 20% in discectomy patients at three and six months to 10% at nine months. This drastic reduction in the percentage was due to a small number of discectomy subjects and it would be too premature to comment on the reduction of incidence over the follow-up period. This calls for further research in the field of instability to study if surgery had any effect on the mobility of L5-S1 segment.

## Conclusions

The quantification of normal and abnormal spinal motion is likely to be still dependent on imaging. If patients with spondylolisthesis were excluded from the study, instability still existed in patients with low back pain mainly because of rotational component in sagittal plane. Younger subjects had greater angular rotations and it decreased as the age progressed. Determination of the relationship between imaging instability and its symptoms remains challenging if not impossible. We did not find any significant correlation between spinal signs and symptoms considered and radiographic instability.

Standard surgical steps performed in lumbar surgeries lead to an occurrence of secondary iatrogenic instabilities. However due to aforementioned limitations including a lesser number of patients, a more detailed study examining all segments separately is necessary to confirm the results and evaluate the development and progression of segmental instability in patients undergoing lumbar surgeries for low back pain.
